# 

**Published:** 1919-10

**Authors:** 


### ANNOUNCEMENTS

NATIONAL ASSOCIATION OF DENTAL FACULTIES. "The annual meeting of the National Association of Dental Faculties will be held in Parlor E, Mezzanine Floor, The Grunewald Hotel, New Orleans. The meeting will be called on Saturday, October 18, at 9 a. m., and will last through Monday."

Very respectfully,

C. C. ALLEN, Secretary.

---

NEW YORK STATE EXAMINATIONS will be held this year the second Tuesday of November, instead of as in former years.

---

NEW JERSEY STATE EXAMINATIONS will be held December 1st in Trenton, N. J.

---

### AMERICAN RED CROSS

HOW YOU CAN HELP! THIRD RED CROSS ROLL CALL NOVEMBER 2 TO 11—

By helping to enroll one million volunteers to bring in the 20,000,000 new members.

By spreading the news of the great public health service campaign, international in its scope.

By helping to interest large organizations in making more efficient their departments of first-aid.

By interesting women in their opportunities to learn home nursing and care of the sick under the Red Cross and thus guard against serious illness and unnecessary loss of life.

By telling women of the free Red Cross cooking courses, where they can learn how to prepare inexpensive nourishing food for their families.

By practicing the helping hand doctrine—the foundation of the Red Cross Home Service.

